# 1,4-Bis{4-[bis­(prop-2-yn-1-yl)amino]­phen­oxy}benzene

**DOI:** 10.1107/S1600536811003862

**Published:** 2011-02-05

**Authors:** Kiramat Shah, M. Raza Shah, Seik Weng Ng

**Affiliations:** a H.E.J. Research Institute of Chemistry, International Center for Chemical and Biological Sciences, University of Karachi, Karachi 7527, Pakistan; b Department of Chemistry, University of Malaya, 50603 Kuala Lumpur, Malaysia

## Abstract

The asymmetric unit of the title compound, C_30_H_24_N_2_O_2_, contains two independent mol­ecules, which both lie on centers of inversion. The central phenyl­ene ring is inclined at 61.4 (2)° with respect to the flanking aromatic ring [dihedral angle = 70.7 (3)° in the second mol­ecule].

## Related literature

For the only reported crystal structure of a compound possessing a propylyl­amino unit, see: Steiner *et al.* (1999 ▶). For the structure of 1,4-bis­(4-amino­phenoxl)benzene, see: Shemsi *et al.* (2008 ▶).
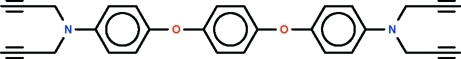


## Experimental

### 

#### Crystal data

C_30_H_24_N_2_O_2_
*M* *_r_* = 444.51
Triclinic, 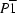

*a* = 9.8766 (7) Å
*b* = 11.1635 (6) Å
*c* = 12.1531 (9) Å
α = 68.687 (6)°
β = 69.601 (7)°
γ = 88.529 (5)°
*V* = 1162.19 (13) Å^3^
*Z* = 2
Mo *K*α radiation
μ = 0.08 mm^−1^
*T* = 100 K
0.30 × 0.10 × 0.05 mm

#### Data collection

Agilent SuperNova Dual diffractometer with an Atlas detector
Absorption correction: multi-scan (*CrysAlis PRO*; Agilent, 2010 ▶) *T*_min_ = 0.712, *T*_max_ = 1.000
9192 measured reflections
5142 independent reflections
3245 reflections with *I* > 2σ(*I*)
*R*_int_ = 0.051

#### Refinement

*R*[*F* ^2^ > 2σ(*F* ^2^)] = 0.063
*wR*(*F* ^2^) = 0.172
*S* = 1.06
5142 reflections
323 parameters
4 restraints
H atoms treated by a mixture of independent and constrained refinement
Δρ_max_ = 0.24 e Å^−3^
Δρ_min_ = −0.33 e Å^−3^

### 

Data collection: *CrysAlis PRO* (Agilent, 2010 ▶); cell refinement: *CrysAlis PRO*; data reduction: *CrysAlis PRO*; program(s) used to solve structure: *SHELXS97* (Sheldrick, 2008 ▶); program(s) used to refine structure: *SHELXL97* (Sheldrick, 2008 ▶); molecular graphics: *X-SEED* (Barbour, 2001 ▶); software used to prepare material for publication: *publCIF* (Westrip, 2010 ▶).

## Supplementary Material

Crystal structure: contains datablocks global, I. DOI: 10.1107/S1600536811003862/jh2263sup1.cif

Structure factors: contains datablocks I. DOI: 10.1107/S1600536811003862/jh2263Isup2.hkl

Additional supplementary materials:  crystallographic information; 3D view; checkCIF report

## Figures and Tables

**Fig. 1.**
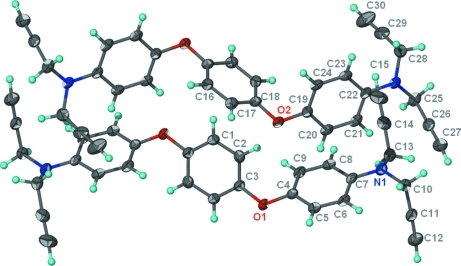
Thermal ellipsoid plot (Barbour, 2001) of C30H24N2O2 at the 70% probability level; hydrogen atoms are drawn as spheres of arbitrary radius.

## supplementary crystallographic information

### Comment

1,4-Bis(4-aminophenoxy)benzene is a precusor for the synthesis of polyamides owing the functional amino –NH_2_ group that will condense with carboxylic acids (Shemsi *et al.*, 2008). The amino group can be also converted to a dialkylamino group by reaction with an alkyl halide in the presence of potassium carbonate. This strategy is used for the synthesis of the nitrogen–propargyl bond. The unit cell has two independent molecules of C_30_H_24_N_2_O_2_ (Scheme I) that both lie on a center-of-inversion (Fig. 1). The central phenylene ring is aligned at 61.4 (2) ° with respect to the flanking aromatic ring (the dihedral angle is 70.7 (3) ° for the second molecule). There is only one reported example of the nitrogen-parpargyl bond (Steiner *et al.*, 1999).

### Experimental

1,4-Bis(4-aminophenoxy)benzene (1 g, 2.2 mmol) was dissolved in ethanl (30 ml) followed by the addition of potassium carbonate (3 g, 21 mmol). The mixture was heated for 1 h. Propargyl bromide (1.5 ml, 15 mmol) was added and the heating was continued for another 8 h. The solvent was evaporated under reduced pressure and the residue was dissolved in a mixture of water (50 ml) and dichloromethane (50 ml). The aqueous layer was extracted three times with dichloromethane and concentrated. The product was recrystallized from ethanol; yield 60%.

### Refinement

Carbon-bound H-atoms were placed in calculated positions [C—H 0.95 to 0.99 Å, *U*_iso_(H) 1.2*U*_eq_(C)] and were included in the refinement in the riding model approximation.

The acetylenic H-atoms were located in a difference Fourier map, and were refined with a distance restraint of C–H 0.95±0.01 Å; their temperature factors were refined.

### Crystal data

**Table:**

C_30_H_24_N_2_O_2_	*Z* = 2
*M**_r_* = 444.51	*F*(000) = 468
Triclinic, *P*1	*D*_x_ = 1.270 Mg m^−^^3^
Hall symbol: -P 1	Mo *K*α radiation, λ = 0.71073 Å
*a* = 9.8766 (7) Å	Cell parameters from 2448 reflections
*b* = 11.1635 (6) Å	θ = 2.2–29.2°
*c* = 12.1531 (9) Å	µ = 0.08 mm^−^^1^
α = 68.687 (6)°	*T* = 100 K
β = 69.601 (7)°	Prism, colorless
γ = 88.529 (5)°	0.30 × 0.10 × 0.05 mm
*V* = 1162.19 (13) Å^3^	

### Data collection

**Table:**

Agilent SuperNova Dual diffractometer with an Atlas detector	5142 independent reflections
Radiation source: SuperNova (Mo) X-ray Source	3245 reflections with *I* > 2σ(*I*)
Mirror	*R*_int_ = 0.051
Detector resolution: 10.4041 pixels mm^-1^	θ_max_ = 27.5°, θ_min_ = 2.2°
ω scans	*h* = −12→12
Absorption correction: multi-scan (*CrysAlis PRO*; Agilent, 2010)	*k* = −12→14
*T*_min_ = 0.712, *T*_max_ = 1.000	*l* = −13→15
9192 measured reflections	

### Refinement

**Table:**

Refinement on *F*^2^	Primary atom site location: structure-invariant direct methods
Least-squares matrix: full	Secondary atom site location: difference Fourier map
*R*[*F*^2^ > 2σ(*F*^2^)] = 0.063	Hydrogen site location: inferred from neighbouring sites
*wR*(*F*^2^) = 0.172	H atoms treated by a mixture of independent and constrained refinement
*S* = 1.06	*w* = 1/[σ^2^(*F*_o_^2^) + (0.0626*P*)^2^] where *P* = (*F*_o_^2^ + 2*F*_c_^2^)/3
5142 reflections	(Δ/σ)_max_ = 0.001
323 parameters	Δρ_max_ = 0.24 e Å^−^^3^
4 restraints	Δρ_min_ = −0.33 e Å^−^^3^

### Fractional atomic coordinates and isotropic or equivalent isotropic displacement parameters (Å^2^)

**Table:**

	*x*	*y*	*z*	*U*_iso_*/*U*_eq_	
O1	0.51131 (17)	0.20860 (14)	0.78479 (13)	0.0252 (4)	
O2	1.00539 (17)	0.19216 (14)	0.77617 (13)	0.0247 (4)	
N1	0.7243 (2)	0.72196 (18)	0.64767 (16)	0.0242 (5)	
N2	1.2235 (2)	0.70018 (18)	0.65456 (17)	0.0240 (5)	
C1	0.6093 (2)	−0.0128 (2)	1.0493 (2)	0.0223 (5)	
H1	0.6838	−0.0217	1.0835	0.027*	
C2	0.6174 (2)	0.0957 (2)	0.9431 (2)	0.0243 (5)	
H2	0.6977	0.1611	0.9038	0.029*	
C3	0.5073 (2)	0.1073 (2)	0.89530 (19)	0.0211 (5)	
C4	0.5691 (2)	0.3332 (2)	0.7564 (2)	0.0212 (5)	
C5	0.6303 (2)	0.4110 (2)	0.6295 (2)	0.0220 (5)	
H5	0.6387	0.3760	0.5671	0.026*	
C6	0.6796 (2)	0.5397 (2)	0.5924 (2)	0.0216 (5)	
H6	0.7206	0.5927	0.5047	0.026*	
C7	0.6698 (2)	0.5927 (2)	0.68269 (19)	0.0205 (5)	
C8	0.6062 (2)	0.5121 (2)	0.8109 (2)	0.0225 (5)	
H8	0.5976	0.5460	0.8739	0.027*	
C9	0.5554 (2)	0.3838 (2)	0.8474 (2)	0.0232 (5)	
H9	0.5114	0.3308	0.9348	0.028*	
C10	0.7819 (3)	0.8070 (2)	0.5142 (2)	0.0249 (5)	
H10A	0.8515	0.7620	0.4663	0.030*	
H10B	0.8367	0.8851	0.5052	0.030*	
C11	0.6713 (3)	0.8483 (2)	0.4560 (2)	0.0257 (5)	
C12	0.5809 (3)	0.8773 (3)	0.4114 (2)	0.0346 (6)	
C13	0.6654 (3)	0.7857 (2)	0.7376 (2)	0.0264 (6)	
H13A	0.5603	0.7558	0.7841	0.032*	
H13B	0.6760	0.8803	0.6901	0.032*	
C14	0.7383 (3)	0.7593 (2)	0.8296 (2)	0.0280 (6)	
C15	0.7930 (3)	0.7348 (3)	0.9058 (3)	0.0416 (7)	
C16	1.0318 (2)	−0.1219 (2)	1.0003 (2)	0.0219 (5)	
H16	1.0540	−0.2052	1.0006	0.026*	
C17	1.0369 (2)	−0.0226 (2)	0.8889 (2)	0.0231 (5)	
H17	1.0618	−0.0381	0.8128	0.028*	
C18	1.0058 (2)	0.0986 (2)	0.88900 (19)	0.0212 (5)	
C19	1.0600 (2)	0.3185 (2)	0.74796 (19)	0.0212 (5)	
C20	0.9869 (2)	0.4191 (2)	0.69923 (19)	0.0236 (5)	
H20	0.9004	0.4016	0.6876	0.028*	
C21	1.0402 (2)	0.5458 (2)	0.6673 (2)	0.0244 (5)	
H21	0.9900	0.6148	0.6333	0.029*	
C22	1.1676 (2)	0.5732 (2)	0.68473 (19)	0.0204 (5)	
C23	1.2417 (2)	0.4694 (2)	0.73047 (19)	0.0220 (5)	
H23	1.3296	0.4859	0.7406	0.026*	
C24	1.1894 (2)	0.3433 (2)	0.76112 (19)	0.0222 (5)	
H24	1.2416	0.2740	0.7910	0.027*	
C25	1.1369 (3)	0.8061 (2)	0.6209 (2)	0.0268 (6)	
H25A	1.1674	0.8791	0.6381	0.032*	
H25B	1.0332	0.7765	0.6748	0.032*	
C26	1.1529 (2)	0.8513 (2)	0.4870 (2)	0.0239 (5)	
C27	1.1667 (3)	0.8879 (2)	0.3791 (2)	0.0323 (6)	
C28	1.3208 (3)	0.7176 (2)	0.7156 (2)	0.0273 (6)	
H28A	1.3485	0.8115	0.6881	0.033*	
H28B	1.4106	0.6782	0.6865	0.033*	
C29	1.2574 (3)	0.6603 (2)	0.8560 (2)	0.0308 (6)	
C30	1.2069 (4)	0.6076 (3)	0.9671 (3)	0.0454 (8)	
H12	0.506 (2)	0.895 (3)	0.377 (2)	0.055 (9)*	
H15	0.838 (3)	0.720 (3)	0.966 (2)	0.055 (9)*	
H27	1.177 (3)	0.911 (3)	0.2929 (12)	0.059 (9)*	
H30	1.167 (3)	0.570 (3)	1.0577 (10)	0.075 (11)*	

### Atomic displacement parameters (Å^2^)

**Table:**

	*U*^11^	*U*^22^	*U*^33^	*U*^12^	*U*^13^	*U*^23^
O1	0.0315 (9)	0.0200 (8)	0.0219 (9)	0.0000 (7)	−0.0140 (7)	−0.0015 (6)
O2	0.0334 (9)	0.0205 (9)	0.0209 (9)	0.0019 (7)	−0.0148 (7)	−0.0039 (6)
N1	0.0316 (11)	0.0219 (10)	0.0180 (10)	0.0034 (9)	−0.0101 (8)	−0.0054 (8)
N2	0.0283 (11)	0.0203 (10)	0.0230 (10)	0.0053 (9)	−0.0135 (9)	−0.0039 (8)
C1	0.0214 (12)	0.0224 (12)	0.0256 (12)	0.0055 (10)	−0.0126 (10)	−0.0082 (9)
C2	0.0210 (12)	0.0222 (12)	0.0249 (13)	−0.0025 (10)	−0.0069 (10)	−0.0049 (9)
C3	0.0226 (12)	0.0210 (12)	0.0184 (12)	0.0029 (10)	−0.0088 (10)	−0.0046 (9)
C4	0.0198 (11)	0.0215 (12)	0.0211 (12)	0.0029 (10)	−0.0100 (9)	−0.0043 (9)
C5	0.0223 (12)	0.0249 (13)	0.0209 (12)	0.0063 (10)	−0.0095 (10)	−0.0099 (9)
C6	0.0223 (12)	0.0245 (12)	0.0153 (11)	0.0063 (10)	−0.0068 (9)	−0.0049 (9)
C7	0.0195 (11)	0.0203 (12)	0.0197 (12)	0.0059 (10)	−0.0094 (9)	−0.0035 (9)
C8	0.0222 (12)	0.0275 (13)	0.0201 (12)	0.0089 (10)	−0.0105 (10)	−0.0094 (9)
C9	0.0209 (12)	0.0263 (13)	0.0169 (12)	0.0039 (10)	−0.0061 (9)	−0.0029 (9)
C10	0.0272 (13)	0.0220 (12)	0.0232 (12)	0.0003 (10)	−0.0094 (10)	−0.0057 (9)
C11	0.0305 (14)	0.0217 (12)	0.0215 (12)	0.0025 (11)	−0.0099 (10)	−0.0038 (9)
C12	0.0382 (16)	0.0354 (15)	0.0340 (15)	0.0100 (13)	−0.0195 (13)	−0.0115 (12)
C13	0.0298 (13)	0.0209 (12)	0.0283 (13)	0.0044 (11)	−0.0116 (11)	−0.0081 (10)
C14	0.0234 (13)	0.0341 (14)	0.0246 (13)	−0.0012 (11)	−0.0039 (10)	−0.0135 (11)
C15	0.0351 (16)	0.061 (2)	0.0308 (15)	−0.0072 (14)	−0.0113 (13)	−0.0200 (14)
C16	0.0236 (12)	0.0189 (12)	0.0252 (12)	0.0062 (10)	−0.0110 (10)	−0.0090 (9)
C17	0.0234 (12)	0.0268 (13)	0.0193 (12)	0.0038 (10)	−0.0070 (10)	−0.0099 (10)
C18	0.0179 (11)	0.0254 (13)	0.0193 (12)	0.0019 (10)	−0.0088 (9)	−0.0051 (9)
C19	0.0246 (12)	0.0211 (12)	0.0148 (11)	−0.0003 (10)	−0.0057 (9)	−0.0045 (9)
C20	0.0198 (12)	0.0291 (13)	0.0184 (12)	0.0035 (10)	−0.0086 (9)	−0.0036 (9)
C21	0.0241 (12)	0.0255 (13)	0.0194 (12)	0.0085 (10)	−0.0092 (10)	−0.0032 (9)
C22	0.0213 (12)	0.0215 (12)	0.0135 (11)	0.0029 (10)	−0.0054 (9)	−0.0021 (9)
C23	0.0193 (12)	0.0278 (13)	0.0195 (12)	0.0056 (10)	−0.0096 (9)	−0.0073 (9)
C24	0.0232 (12)	0.0254 (13)	0.0180 (12)	0.0072 (10)	−0.0099 (9)	−0.0061 (9)
C25	0.0311 (14)	0.0238 (13)	0.0223 (12)	0.0069 (11)	−0.0085 (10)	−0.0065 (10)
C26	0.0216 (12)	0.0218 (12)	0.0269 (13)	0.0054 (10)	−0.0092 (10)	−0.0074 (10)
C27	0.0332 (15)	0.0343 (15)	0.0312 (16)	0.0047 (12)	−0.0151 (12)	−0.0114 (12)
C28	0.0250 (13)	0.0241 (13)	0.0332 (14)	0.0040 (10)	−0.0126 (11)	−0.0093 (10)
C29	0.0367 (14)	0.0318 (14)	0.0358 (16)	0.0162 (12)	−0.0211 (12)	−0.0193 (12)
C30	0.067 (2)	0.0478 (18)	0.0350 (18)	0.0323 (16)	−0.0280 (16)	−0.0231 (14)

### Geometric parameters (Å, °)

**Table:**

O1—C4	1.391 (3)	C13—C14	1.472 (3)
O1—C3	1.395 (2)	C13—H13A	0.9900
O2—C18	1.391 (2)	C13—H13B	0.9900
O2—C19	1.399 (3)	C14—C15	1.175 (3)
N1—C7	1.410 (3)	C15—H15	0.95 (1)
N1—C10	1.457 (3)	C16—C18^ii^	1.384 (3)
N1—C13	1.463 (3)	C16—C17	1.389 (3)
N2—C22	1.404 (3)	C16—H16	0.9500
N2—C28	1.458 (3)	C17—C18	1.380 (3)
N2—C25	1.461 (3)	C17—H17	0.9500
C1—C3^i^	1.378 (3)	C18—C16^ii^	1.384 (3)
C1—C2	1.391 (3)	C19—C20	1.380 (3)
C1—H1	0.9500	C19—C24	1.388 (3)
C2—C3	1.384 (3)	C20—C21	1.388 (3)
C2—H2	0.9500	C20—H20	0.9500
C3—C1^i^	1.378 (3)	C21—C22	1.405 (3)
C4—C5	1.379 (3)	C21—H21	0.9500
C4—C9	1.381 (3)	C22—C23	1.399 (3)
C5—C6	1.385 (3)	C23—C24	1.382 (3)
C5—H5	0.9500	C23—H23	0.9500
C6—C7	1.399 (3)	C24—H24	0.9500
C6—H6	0.9500	C25—C26	1.467 (3)
C7—C8	1.400 (3)	C25—H25A	0.9900
C8—C9	1.385 (3)	C25—H25B	0.9900
C8—H8	0.9500	C26—C27	1.181 (3)
C9—H9	0.9500	C27—H27	0.950 (10)
C10—C11	1.477 (3)	C28—C29	1.481 (3)
C10—H10A	0.9900	C28—H28A	0.9900
C10—H10B	0.9900	C28—H28B	0.9900
C11—C12	1.180 (3)	C29—C30	1.178 (4)
C12—H12	0.96 (1)	C30—H30	0.956 (10)
			
C4—O1—C3	120.48 (16)	C14—C13—H13B	109.1
C18—O2—C19	116.42 (16)	H13A—C13—H13B	107.8
C7—N1—C10	119.60 (18)	C15—C14—C13	177.7 (3)
C7—N1—C13	118.73 (18)	C14—C15—H15	177.1 (18)
C10—N1—C13	115.86 (18)	C18^ii^—C16—C17	119.6 (2)
C22—N2—C28	117.61 (17)	C18^ii^—C16—H16	120.2
C22—N2—C25	119.25 (19)	C17—C16—H16	120.2
C28—N2—C25	116.15 (19)	C18—C17—C16	119.9 (2)
C3^i^—C1—C2	119.6 (2)	C18—C17—H17	120.0
C3^i^—C1—H1	120.2	C16—C17—H17	120.0
C2—C1—H1	120.2	C17—C18—C16^ii^	120.50 (19)
C3—C2—C1	119.4 (2)	C17—C18—O2	117.25 (19)
C3—C2—H2	120.3	C16^ii^—C18—O2	122.1 (2)
C1—C2—H2	120.3	C20—C19—C24	120.5 (2)
C1^i^—C3—C2	121.0 (2)	C20—C19—O2	118.4 (2)
C1^i^—C3—O1	115.80 (19)	C24—C19—O2	121.0 (2)
C2—C3—O1	123.04 (19)	C19—C20—C21	119.8 (2)
C5—C4—C9	120.1 (2)	C19—C20—H20	120.1
C5—C4—O1	116.31 (19)	C21—C20—H20	120.1
C9—C4—O1	123.34 (19)	C20—C21—C22	120.8 (2)
C4—C5—C6	120.4 (2)	C20—C21—H21	119.6
C4—C5—H5	119.8	C22—C21—H21	119.6
C6—C5—H5	119.8	C23—C22—N2	119.9 (2)
C5—C6—C7	120.7 (2)	C23—C22—C21	118.0 (2)
C5—C6—H6	119.7	N2—C22—C21	122.11 (19)
C7—C6—H6	119.7	C24—C23—C22	121.2 (2)
C6—C7—C8	117.8 (2)	C24—C23—H23	119.4
C6—C7—N1	121.92 (19)	C22—C23—H23	119.4
C8—C7—N1	120.2 (2)	C23—C24—C19	119.6 (2)
C9—C8—C7	121.2 (2)	C23—C24—H24	120.2
C9—C8—H8	119.4	C19—C24—H24	120.2
C7—C8—H8	119.4	N2—C25—C26	112.17 (19)
C4—C9—C8	119.8 (2)	N2—C25—H25A	109.2
C4—C9—H9	120.1	C26—C25—H25A	109.2
C8—C9—H9	120.1	N2—C25—H25B	109.2
N1—C10—C11	114.87 (19)	C26—C25—H25B	109.2
N1—C10—H10A	108.5	H25A—C25—H25B	107.9
C11—C10—H10A	108.5	C27—C26—C25	179.6 (3)
N1—C10—H10B	108.5	C26—C27—H27	175.8 (18)
C11—C10—H10B	108.5	N2—C28—C29	113.99 (19)
H10A—C10—H10B	107.5	N2—C28—H28A	108.8
C12—C11—C10	177.9 (3)	C29—C28—H28A	108.8
C11—C12—H12	176.0 (17)	N2—C28—H28B	108.8
N1—C13—C14	112.7 (2)	C29—C28—H28B	108.8
N1—C13—H13A	109.1	H28A—C28—H28B	107.6
C14—C13—H13A	109.1	C30—C29—C28	176.1 (3)
N1—C13—H13B	109.1	C29—C30—H30	177 (2)
			
C3^i^—C1—C2—C3	0.5 (4)	C18^ii^—C16—C17—C18	0.6 (4)
C1—C2—C3—C1^i^	−0.5 (4)	C16—C17—C18—C16^ii^	−0.6 (4)
C1—C2—C3—O1	−175.8 (2)	C16—C17—C18—O2	−176.80 (19)
C4—O1—C3—C1^i^	146.9 (2)	C19—O2—C18—C17	−142.9 (2)
C4—O1—C3—C2	−37.5 (3)	C19—O2—C18—C16^ii^	41.0 (3)
C3—O1—C4—C5	151.1 (2)	C18—O2—C19—C20	−138.0 (2)
C3—O1—C4—C9	−35.0 (3)	C18—O2—C19—C24	45.9 (3)
C9—C4—C5—C6	0.6 (3)	C24—C19—C20—C21	−2.3 (3)
O1—C4—C5—C6	174.64 (18)	O2—C19—C20—C21	−178.41 (18)
C4—C5—C6—C7	0.8 (3)	C19—C20—C21—C22	−0.4 (3)
C5—C6—C7—C8	−1.3 (3)	C28—N2—C22—C23	−23.4 (3)
C5—C6—C7—N1	177.5 (2)	C25—N2—C22—C23	−173.04 (19)
C10—N1—C7—C6	5.2 (3)	C28—N2—C22—C21	158.4 (2)
C13—N1—C7—C6	157.2 (2)	C25—N2—C22—C21	8.8 (3)
C10—N1—C7—C8	−176.13 (19)	C20—C21—C22—C23	2.4 (3)
C13—N1—C7—C8	−24.1 (3)	C20—C21—C22—N2	−179.4 (2)
C6—C7—C8—C9	0.5 (3)	N2—C22—C23—C24	179.92 (19)
N1—C7—C8—C9	−178.26 (19)	C21—C22—C23—C24	−1.8 (3)
C5—C4—C9—C8	−1.3 (3)	C22—C23—C24—C19	−0.8 (3)
O1—C4—C9—C8	−174.98 (19)	C20—C19—C24—C23	2.9 (3)
C7—C8—C9—C4	0.8 (3)	O2—C19—C24—C23	178.92 (18)
C7—N1—C10—C11	72.2 (3)	C22—N2—C25—C26	−82.9 (2)
C13—N1—C10—C11	−80.6 (3)	C28—N2—C25—C26	127.0 (2)
C7—N1—C13—C14	85.2 (2)	C22—N2—C28—C29	−56.4 (3)
C10—N1—C13—C14	−121.7 (2)	C25—N2—C28—C29	94.1 (2)

Symmetry codes: (i) −*x*+1, −*y*, −*z*+2; (ii) −*x*+2, −*y*, −*z*+2.

### Hydrogen-bond geometry (Å, °)

**Table:**

*D*—H···*A*	*D*—H	H···*A*	*D*···*A*	*D*—H···*A*
C12—H12···O1^iii^	0.96 (1)	2.66 (3)	3.263 (3)	121 (2)
C27—H27···O2^iv^	0.95 (1)	2.68 (2)	3.285 (3)	122 (2)

Symmetry codes: (iii) −*x*+1, −*y*+1, −*z*+1; (iv) −*x*+2, −*y*+1, −*z*+1.
